# Allelic variants of *DYX1C1* are not associated with dyslexia in India

**DOI:** 10.4103/0971-6866.45002

**Published:** 2008

**Authors:** Pushpa Saviour, Satish Kumar, U. Kiran, Rajasekhara Reddy Ravuri, V. R. Rao, Nallur Basappa Ramachandra

**Affiliations:** Human Genetics Laboratory, Department of Studies in Zoology, University of Mysore, Manasagangothri, Mysore-570 006, India; 1 Anthropological Survey of India, Southern Regional Centre, Manav Bhavan, Bogadi II Stage, Mysore-570 006, India

**Keywords:** Candidate gene, chromosome, dyslexia, *DYX1C1*

## Abstract

Dyslexia is a hereditary neurological disorder that manifests as an unexpected difficulty in learning to read despite adequate intelligence, education, and normal senses. The prevalence of dyslexia ranges from 3 to 15% of the school aged children. Many genetic studies indicated that loci on 6p21.3, 15q15-21, and 18p11.2 have been identified as promising candidate gene regions for dyslexia. Recently, it has been suggested that allelic variants of gene, *DYX1C1* influence dyslexia. In the present study, exon 2 and 10 of *DYX1C1* has been analyzed to verify whether these single nucleotide polymorphisms (SNPs) influence dyslexia, in our population. Our study identified 4 SNPs however, none of these SNPS were found to be significantly associated with dyslexia suggesting *DYX1C1* allelic variants are not associated with dyslexia.

## Introduction

Developmental dyslexia is a hereditary neurological disorder that manifests as a persistent difficulty in learning to read and spell in children with otherwise normal intellectual functioning and educational opportunities.[1] Prevalence of dyslexia in school children has been found to be 3-17.5%.[2] The tendency of dyslexia to run in families has become clear since its earliest descriptions and modern family and twin studies indicate that heritability is 50-60%.[3] Molecular linkage studies have indicated different chromosomal areas harboring dyslexia candidate genes on chromosomes 1, 2, 3, 6, 15, and 18.[4–9] However, characterization of candidate genes for dyslexia is still in the infancy. One of the possible candidate genes, which influence dyslexia, is *DYX1C1* which is near *DYX1* locus on chromosome 15q21. Sequence analysis of *DYX1C1* shows eight single nucleotide polymorphisms (SNPs), of them two SNPs, -3G>A and 1249G>T are functionally important and influences dyslexia.[10]

Because SNPs are inherited and do not change much from generation to generation, analysis of SNPs is essential for finding genes that predispose people to more common conditions in which inheritance patterns are complex and also it will have a wide range of applications for developing diagnostic, therapeutic, and preventative strategies. Since dyslexia is a major educational problem, there is a need for detailed genetic analysis to find out the genes which are responsible for dyslexia which in turn will provide simple diagnostic tools to ease the clinicians for early evaluation of the disorder and treatment. In the present investigation, an attempt has been made to verify whether allelic variants of *DYX1C1* are responsible for dyslexia in our population.

## Materials and Methods

Dyslexic children were ascertained through special schools for learning disabled as well as from regular schools of Karnataka state. Following tests were used for the diagnosis: a) Teacher rating: Rutter's Proforma A and B[11] was used to get the teachers rating on children's academic performance as well as the presence of behavioral and emotional problems to eliminate those with severe behavioral/ emotional problems if they are primary causes of poor academic achievement. b) Raven's (Colored) Progressive Matrices (RCPM/RPM) was used to ascertain that those children with poor reading/writing are not below normal in their intellectual/reasoning function.[12] c) Graded reading and spelling tests were administered to ascertain that they were behind at least by two grade norms in reading as required by the operational definition of dyslexia. In addition to the above-mentioned criteria, school examination marks and clinical certificates issued from institutes such as National Institute of Mental Health and Neurosciences, Bangalore and All India Institute of Speech and Hearing, Mysore were used as supportive evidences. The age range of the subjects was 8-17 years. Individuals who had no history of reading, spelling, or other academic difficulties were selected as control subjects.

Genomic DNA was isolated from peripheral blood of 51 control subjects and 52 dyslexic subjects by phenol-chloroform DNA isolation method and subjected for screening of SNPs and mutations in the exon 2 as well as 10 of *DYX1C1* gene. Using exon 2 flanking intronic primers, a total of 477bp were amplified in all the subjects and also using exon 10 flanking intronic primers, a total of 698bp were amplified. The amplified products were subjected for Sanger's DNA sequencing.

## Results

**Figure 1:**
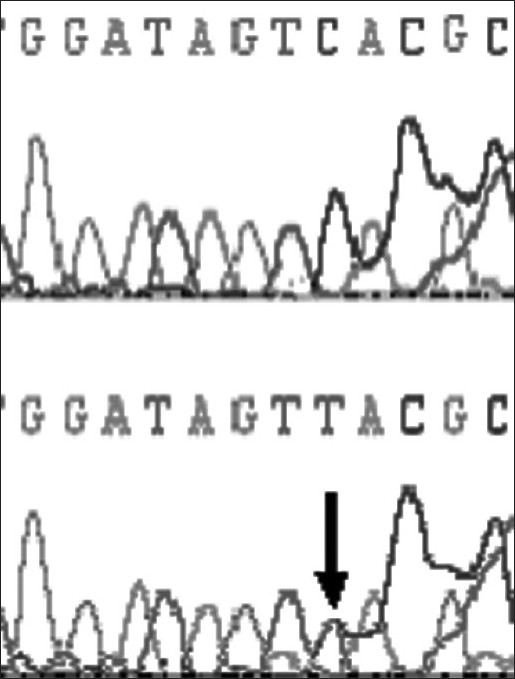
Chromatogram of control sample showing normal sequence and dyslexic sample showing heterozygote (C-T) at -164 position of exon 2 of *DYX1C1* gene (arrow indicates the site)

**Figure 2:**
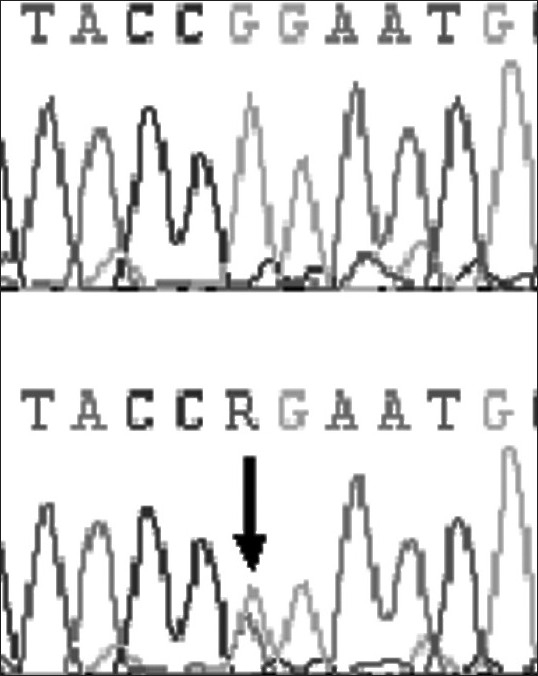
Chromatogram of dyslexic sample showing normal and another sample showing heterozygote (G-A) at -3 position of exon 2 of *DYX1C1* gene (arrow indicates the site). R indicatesG/A

**Figure 3:**
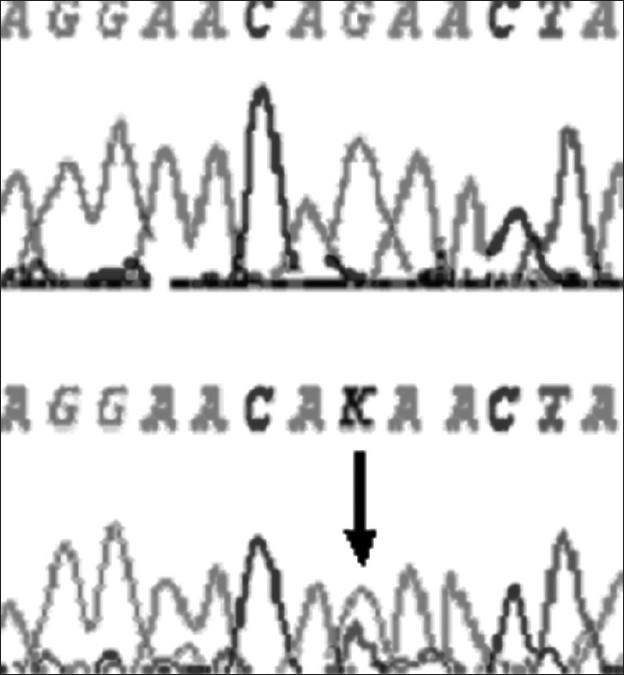
Chromatogram of dyslexic sample showing normal and another sample showing heterozygote (G-T) at 1249 position of exon 10 of *DYX1C1* gene (arrow indicates the site). K indicates G/T

**Figure 4:**
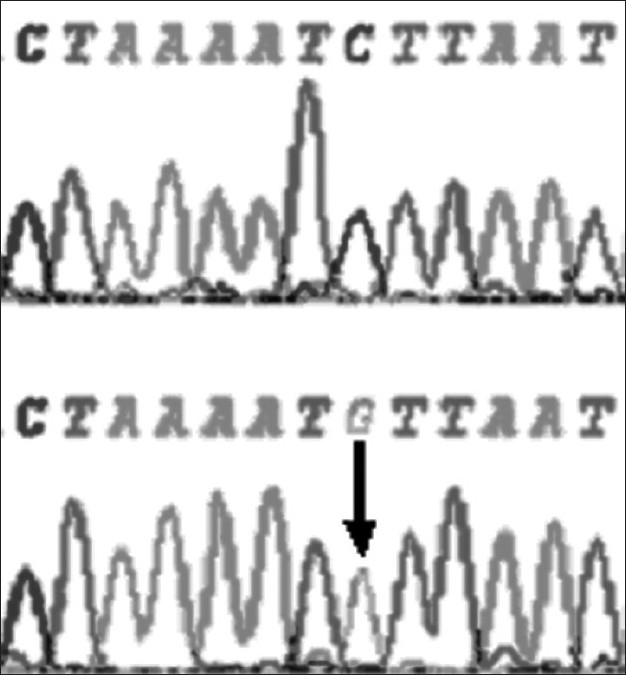
Chromatogram of dyslexic sample showing normal and another sample showing C-G SNP (homozygote) at 1259 position of exon 10 of *DYX1C1* gene (arrow indicates the site)

At-164 position of exon 2 of *DYX1C1,* C to T transversion was observed in one dyslexic proband and none of the controls showed this polymorphism at this site [Figure 1]. At –3 position of exon 2, G to A polymorphism was found in 4 controls and 7 dyslexics [Figure 2]. At 1249 position of 10^th^ exon G to T SNP was found in three dyslexic cases and none of the control sample showed this polymorphism [Figure 3]. At 1259 position of 10^th^ exon, C to G polymorphism was found in 4 controls and 8 dyslexic cases [Figure 4]. All the polymorphisms were in both exons 2 and 10 were heterozygous except the SNP at 1259 position of exon 10. Allele frequencies of these SNPs in dyslexics and control subjects are presented in Table 1. However, chi squire test shows no significant *P* values for these SNPs [Table 1]. Comparison of *DYX1C1* allele frequencies observed in UK, Finnish, and present study is given in Table 2.

**Table 1:** Single nucleotide polymorphisms observed in 2^nd^ and 10^th^ exon in 52 dyslexic cases and 51 controls subjects

Polymorphism	Exon	% of allele frequency		Chi-square value	*P* value
				
		Dyslexics	Controls		
-164 C>T	2	0.96	0	0.986	0.421
-3 G>A	2	6.73	3.92	0.804	0.370
1249 G>T	10	2.88	0	2.986	0.084
1259 C>G	10	7.84	3.9	1.335	0.248

**Table 2:** Comparison of *DYX1C1* allele frequencies observed in UK, Finland, and present study

Sequence	Coding	UK allele (%)	Finnish	Present
variant	change		allele (%)	study (%)
−164C-T	(5′-UTR)	T (1.56)	T (1.0–6.4)	T (0.96)
−3G-A	(5′-UTR)	A (6.37)	A (2.5–8.3)	A (6.73)
1249G-T	Glu417X	T (9.63)	T (5–13.2)	T (2.88)
1259C-G	Ser420Cys	G (9.80)	G (2–10)	G (7.84)

## Discussion

Many genetic studies on dyslexia have identified specific chromosomal loci for different dyslexia related phenotypes which suggest many genes are contributing to the predisposition of dyslexia.[13] One of the possible candidate genes which influence dyslexia is *DYX1C1.* It consists of 10 exons and codes for 420 amino acid protein which is expressed in brain, lung, kidney, and testis. In the brain, it is expressed in white matter glial cells and cortical neuronal cells. Eight SNPs are located in *DYX1C1*, of them two SNPs -3G>A and 1249C>T was reported to be associated with dyslexia. -3G>A is located in the binding sequence of the transcription factors and it was reported that transcriptional activator, Elk-1 has been associated with learning in rats. SNP 1249C>T brings a functional effect by truncating the protein. Thus, it has been suggested that both SNPs are functionally important and influences dyslexia.[10] To verify whether these SNPs are unique across the language, in the present study, exon 2 and exon 10 of *DYX1C1* were amplified and sequenced for SNPs. Our study identified 4 SNPs however, none of these SNPS were found to be significantly associated with dyslexia. Marino *et al*.[14] suggested unitary hypothesis of biological basis of dyslexia. If so, the genes responsible for dyslexia should be universal however, in our study it was found that SNPs, -3G-A and 1249C-T are not functionally important to manifest dyslexia. Reports from Italian and UK population also suggest *DYX1C1* allelic variants are not associated with dyslexia.[15–16] Cellular function of *DYX1C1* is not known so far hence, *DYX1C1* cannot be considered as the candidate gene for dyslexia. Since dyslexia is a complex cognitive disability that affects different aspects of reading related skills which are coordinated by visual, motor, cognitive, and language areas of the brain, it is obvious that dyslexia results from many genetic variants. Most recently a new gene, *ROBO1* is reported near the *DYX5* locus on chromosome 3p which was disrupted due to a translocation t(3;8)(p12;q11), in a dyslexic patient. *ROBO1* is a neuronal axon guidance receptor gene involved in brain development and thus an attractive candidate gene for dyslexia. Two functional copies of *ROBO1* is required in brain development to acquire normal reading development and dyslexia may be caused by partial haplo-insufficiency for *ROBO1.*[17] Another candidate gene for dyslexia is *DCDC2* which is located in the *DYX2* locus and *DCDC2* localizes to the regions of the brain where fluent reading occurs.[18] Though reports of candidate genes of dyslexia are accumulating none of the studies are replicated so far.

Dyslexia is a complex cognitive disability that affects different aspects of reading related skills which are coordinated by visual, motor, cognitive, and language areas of the brain. Thus, dyslexia can result from deviation of normal anatomy and function of those areas in the brain.[9] Studies have identified loci on 6p21.3, 15q15-21, and 18p11.2 as promising candidate gene regions.[8,19–20] Identification of specific risk genes on these regions would help in early diagnosis and once genes have been identified, the study of their gene products and areas of the brain in which they are expressed can shed light on the neurobiological basis of dyslexia.
